# Dissecting protein architecture with communication blocks and communicating segment pairs

**DOI:** 10.1186/s12859-015-0855-y

**Published:** 2016-01-20

**Authors:** Yasaman Karami, Elodie Laine, Alessandra Carbone

**Affiliations:** 1 Sorbonne Universités, UPMC-Univ P6, CNRS, Laboratoire de Biologie Computationnelle et Quantitative - UMR 7238, 15 rue de l’Ecole de Médecine, Paris, 75006 France; 2 Institut Universitaire de France, Paris, 75005 France; 3 Sorbonne Universités, UPMC Univ Paris 06, ICS, Paris, 75005 France

**Keywords:** Protein structure, Protein dynamics, Allostery, Molecular dynamics, Residue network

## Abstract

**Background:**

Proteins adapt to environmental conditions by changing their shape and motions. Characterising protein conformational dynamics is increasingly recognised as necessary to understand how proteins function. Given a conformational ensemble, computational tools are needed to extract in a systematic way pertinent and comprehensive biological information.

**Results:**

Here, we present a method, Communication Mapping (COMMA), to decipher the dynamical architecture of a protein. The method first extracts residue-based dynamic properties from all-atom molecular dynamics simulations. Then, it integrates them in a graph theoretic framework, where it identifies groups of residues or protein regions that mediate short- and long-range communication. COMMA introduces original concepts to contrast the different roles played by these regions, namely *communication blocks* and *communicating segment pairs*, and evaluates the connections and communication strengths between them. We show the utility and capabilities of COMMA by applying it to three archetypal proteins, namely protein A, the tyrosine kinase KIT and the tumour suppressor p53.

**Conclusion:**

Our method permits to compare in a direct way the dynamical behaviour either of proteins with different characteristics or of the same protein in different conditions. It is useful to identify residues playing a key role in protein allosteric regulation and to explain the effects of deleterious mutations in a mechanistic way. COMMA is a fully automated tool with broad applicability. It is freely available to the community at www.lcqb.upmc.fr/COMMA.

**Electronic supplementary material:**

The online version of this article (doi:10.1186/s12859-015-0855-y) contains supplementary material, which is available to authorized users.

## Background

Protein conformational dynamics are directly linked to protein functions [1, 2]. They are sensitive to environmental changes, point mutations, ligand binding and post-translational biochemical modifications [3–5]. Atomistic molecular simulation is a method of choice to explore a protein’s conformational space. It has become increasingly popular with the recent advances in computational power, force field accuracy and sampling algorithm development [6, 7]. The accumulation of molecular dynamics (MD) data calls for the development of methods able to extract pertinent biological information and visualise it in a comprehensive way.

The representation of a protein as a graph unravels more easily and readily its properties at the atomic or residue level. Typically, each node of the graph represents one residue of the protein and the edges represent non-covalent interactions that stabilise the protein three-dimensional structure [8, 9]. Information about the dynamical behaviour of the protein can also be integrated in several ways. For example, the edges can be constructed and weighted based on the persistence values of the interactions computed over a conformational ensemble instead of their presence/absence in a static structure [10]. Other types of dynamic properties can be taken into consideration, such as dynamical correlations between residues [11–13]. Alternatively, every conformation of a MD trajectory can be represented by a contact graph and the evolution of the graphs can be analysed over time to detect important structure-changing events [14].

Communication between residues results in allosteric coupling, i.e. the propagation of a perturbation signal between distinct sites, possibly located far away in the sequence and structure of the protein, that modulates the function of the protein. Experimental evidence have demonstrated that protein residues communicate either through stable non-covalent interactions [15] or via changes in their local atomic fluctuations [16]. Previous methodological efforts were engaged by us and others toward the identification of clusters or chains of residues mediating long-range communication in proteins [17–25]. In particular, the method MONETA [19] proved useful to identify communication routes in allosterically regulated proteins and to guide *in silico* mutagenesis [25]. MONETA is intended to assist the analysis of MD simulation data in a manually-guided way. It enables to focus on specific protein regions or residues provided that the user has some prior knowledge of the system. Fixed values are encoded in the tool for most of the parameters, which limits its applicability and flexibility.

The present work builds up on these previous efforts to propose a systematic dissection of protein architectures from a dynamical perspective. We provide Communication Mapping (COMMA), a method for analysing molecular dynamics-based communication in proteins and for mapping this information onto protein three-dimensional structures. COMMA introduces new measures and new algorithms, with respect to MONETA, to dissect a protein’s architecture building blocks. It integrates different types of structural and dynamical information in a unified graph representing the protein. It detects communication blocks and communicating segments pairs from this graph, which are new concepts representing groups of residues or protein regions that mediate short- and long-range communication. COMMA allows to compare in a very straightforward way the conformational dynamics of different proteins or different states of the same protein. It provides mechanistic insights on the effects of deleterious mutations on the stability and internal dynamics of proteins by pinpointing residues playing key roles in the propagation of these effects. COMMA is fully automated and is intended for large-scale application. It only requires an ensemble of protein conformations as input. Importantly, we have implemented an automated procedure to set all parameters depending on the properties of the protein analysed. Here, we have applied COMMA on three case studies to illustrate its capabilities.

## Methods

### COMMA workflow

**Fig. 1:**
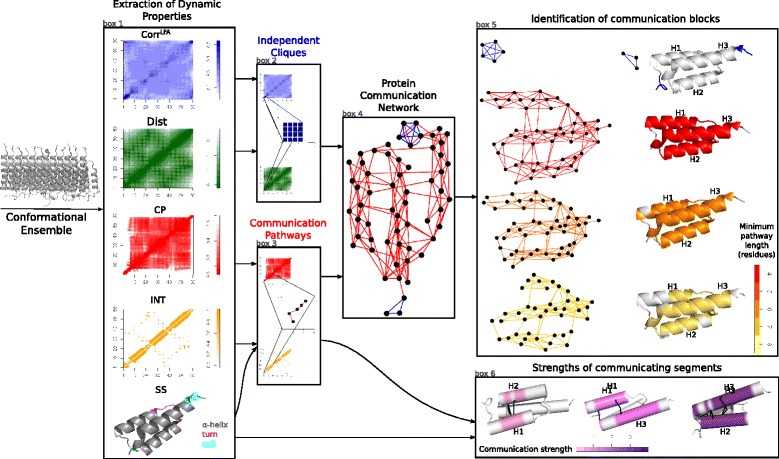
Schematic representation of COMMA workflow. Starting from one or several MD trajectories, COMMA computes matrices of residue-based dynamic properties: local dynamical correlations (Corr ^*LFA*^), minimum distances (Dist), communication propensities (CP), non-covalent interaction strengths (INT) and secondary structures (SS). Local dynamical correlations and minimum distances are used to identify independent cliques while communication propensities, non-covalent interaction strengths and secondary structures are used for communication pathway detection. A coloured graph, called Protein Communication Network (PCN), is constructed from independent cliques (blue edges) and communication pathways (red edges). The graph is analysed and two groups of communication blocks are extracted. The first group is made of clique-based blocks (blue cliques in PCN), and the second group is made of pathway-based blocks (subgraphs of the red PCN) where pathways have bounded length. In the schema, three different pathway-based blocks are displayed, corresponding to a minimal path length of 4 (red), 8 (orange) and 9 (yellow) respectively. Communication pathways are also used to detect pairs of communicating segments, which are portions of secondary structure elements. Residues belonging to pathways that cross two secondary structures are coloured. For each pair of segments, the communication strength of the interaction is evaluated, on a scale of strengths going from low (pink) to strong (violet) strength. The segments and their interaction strength between H1-H2, H1-H3 and H2-H3 helices are shown
- *b.* These properties are used to group residues into (*i*) independent cliques and (*ii*) communication pathways (boxes 2–3). Independent cliques are clusters of residues that display concerted atomic fluctuations while communication pathways are non-covalent chains of residues that move together (see below).
- *c.* The information obtained from the independent cliques and the communication pathways is integrated in a graph, called Protein Communication Network (PCN) (box 4).
- *d.* Connected components are extracted from this graph to define protein communication blocks (box 5).
- *e.* The communication pathways that link different secondary structure elements are used to define communicating segment pairs and measure the strength of the interaction (box 6).

The workflow of the COMMA method is depicted on Fig. 1. COMMA requires as input a conformational ensemble representing the protein of interest. Typically, the method is intended to analyse all-atom MD trajectories, but it is not restricted to this type of data. The analysis can also be performed on conformations obtained from another sampling method or on experimentally determined structures. The order of the input conformations does not influence the results. The ensemble can be divided into several sets, for example corresponding to several replicates of an MD simulation. COMMA can handle most popular MD trajectory file formats (Table 2). COMMA algorithm proceeds as follows:

- *a.* It analyses the conformational ensemble and extracts five residue-based dynamic properties: local dynamical correlations, minimum distances, communication propensities, non-covalent interaction strengths and secondary structures (box 1).

COMMA allows to visualise communication blocks and communicating segment pairs by mapping them onto the protein average conformation.

### Step a. Extraction of dynamic properties

COMMA defines several measures that reflect the dynamic properties of the query protein. These measures are computed from each input set of conformations. Four measures are defined for pairs of residues and provide 4 distinct matrices. A fifth measure, which is new compared to MONETA, evaluates the likeliness of a residue to belong to a secondary structure.

*Local dynamical correlations* Principal Component Analysis (PCA) is used to describe the atomic fluctuations of a protein through eigenvectors or modes. These modes are linear combinations of degrees of freedom. Starting from *n* PCA modes, describing the protein’s essential dynamics (i.e. explaining 80 % of the total atomic fluctuations), we apply a statistical technique called Local Feature Analysis (LFA) [26]. LFA computes residual correlations *Corr*^*LFA*^(*i,j*) between residues *i* an *j* as:

(1) $$ Corr^{LFA}(i,j)= \sum\limits_{d=1}^{3} \sum\limits_{r=1}^{n} \Psi_{r}(i_{d}) \Psi_{r}(j_{d})  $$

where *d* is the (*x,y,z*)-coordinate index of each C *α* atom in a residue and *Ψ*_*r*_ is the PCA *r*^*th*^ eigenvector. The *Corr*^*LFA*^ matrix is characterised by sparse correlation patterns (see on Fig. 1). The LFA formalism identifies a set of *nseed* residues that are highly fluctuating and representative of these correlation patterns.

*Minimum distances* The minimum distance $d^{min}_{\textit {ij}}$ between two residues *i* and *j* is defined as the smallest distance between any pair of atoms (*a*_*i*_, *a*_*j*_) belonging to residues *i* and *j* respectively, averaged over the set of conformations.

*Communication propensities* We evaluate the communication propensity *CP*(*i,j*) of residues *i* and *j* as the variance of the inter-residue distance [27]:

(2) $$ CP(i,j)\ =\ <(d_{ij}-\bar{d_{ij}})^{2}>$$

where *d*_*ij*_ is the distance between the C *α* atoms of residues *i* and *j* and $\bar {d_{\textit {ij}}}$ is the mean value computed over the set of conformations. Intuitively, the smaller the variance, the more efficient the communication. Consequently, small values of *CP*(*i,j*) are indicative of efficient signal transmission between residues *i* and *j*.

*Non-covalent interaction strengths* We consider as non-covalent interactions hydrogen(H)-bonds and hydrophobic contacts, detected using the HBPLUS algorithm [28]. H-bonds are detected between donor (D) and acceptor (A) atoms that satisfy the following geometric criteria: (*i*) maximum distances of 3.9Å for D-A and 2.5Å for H-A, (*ii*) minimum value of 90° for D-H-A, H-A-AA and D-A-AA angles, where AA is the acceptor antecedent. Hydrophobic contacts are identified with an inter-atomic distance lower than 3.9Å. The detected non-covalent interactions are then classified as backbone-backbone, backbone-side chain and side chain-side chain. For a given interaction type, an interaction strength matrix *INT* is computed, where each entry (*i,j*) describes the percentage of conformations in which at least one non-covalent interaction is formed between some pair of atoms (*a*_*i*_, *a*_*j*_) in residues *i* and *j*.

*Secondary structures* Secondary structures are defined from the backbone torsion angles of the protein by using the DSSP algorithm [29]. Three persistence values *p*_*α*_, *p*_*β*_ and *p*_*turn*_ are computed for each residue. They reflect the percentage of conformations in which the residue is in a *α*-helix, a *β*-sheet or a turn, respectively. The secondary structure type that has the highest persistence value is assigned to the residue.

### Step b. Identification of independent cliques and communication pathways

By combining the measures described above, COMMA identifies groups of residues that mediate communication across the protein structure, namely independent cliques and communications pathways. The computation is performed on each input set of conformations. These components are similar to the independent dynamic segments and communication pathways identified by MONETA. What is new in COMMA is the automated set up of pertinent values for the parameters depending on the system studied (see Parameters).

#### Independent cliques

It can happen that two seeds detected by LFA are very close in the sequence (distant by less than 6 residues). In that case, only the seed with the highest fluctuations is retained. The *Corr*^*LFA*^ matrix is characterised by dense correlation patterns around every seed identified by LFA analysis. COMMA defines independent cliques as protein regions that correspond to these patterns. Each seed is extended into an independent clique *S* of residues by means of an extension algorithm that progressively adds residues in such a way that: (i) have a minimum distance smaller than 3.7Å and (ii) display concerted atomic fluctuations, indicated by high local dynamical correlations, that is the mean correlation value computed over *S* must be higher than a threshold [25]:

(3) $$ \frac{1}{|S|} \sum\limits_{i,j\epsilon S} Corr^{LFA}(i,j) \geq Corr^{LFA}_{cut}  $$

The set up of $Corr^{LFA}_{\textit {cut}}$ is explained below (see Parameters). The extension algorithm terminates when no more residue can be added. At the beginning of the iteration, *S* is made by the starting seed. We obtain *k*≤*n* independent cliques, where *n* is the initial number of seeds. Notice that the algorithm identifying the independent cliques uses information coming from the local dynamical correlation and the minimum distance matrices.

#### Communication pathways

Any two residues *i* and *j* are considered to communicate efficiently if their communication propensity is below a threshold, *CP*(*i,j*)≤*CP*_*cut*_. They form stable non-covalent interaction(s) if their interaction strength is higher than a threshold, *INT*(*i,j*)≥*INT*_*cut*_. The set up of the parameters *CP*_*cut*_ and *INT*_*cut*_ is explained below (see Parameters). Starting from a given residue, the algorithm implemented in COMMA generates a tree of paths that satisfies the following conditions [25]: two consecutive residues in a path (*i*) are not adjacent in the sequence, (*ii*) form stable non-covalent interaction(s) and (*iii*) communicate efficiently. We ask that all residues in a path communicate efficiently with each other by transitivity. Notice that the algorithm identifying the pathway-based edges uses the communication propensity and the interaction strength matrices, and also the secondary structure information, that plays a role for the set up of *CP*_*cut*_ (see Parameters).

### Step c. Construction of a protein communication network

Independent cliques and communication pathways are used to construct a Protein Communication Network (PCN) that reflects the way information is transmitted across the protein 3D structure. A PCN(*N,E*) is a coloured graph defined by nodes *N* that correspond to the residues of the protein and edges *E* that connect dynamically correlated residues. Two types of edges are constructed:

1. **Clique-based edges:** two vertices representing residues *i* and *j* are connected by a clique-based edge if they belong to the same independent clique and if $Corr^{LFA}(i,j) \geq Corr^{LFA}_{\textit {cut}}$.
2. **Pathway-based edges:** two vertices representing residues *i* and *j* are connected by a pathway-based edge if they are consecutive in some communication pathway.

The PCN is constructed by considering the union of all independent cliques and all communication pathways detected from every input set of conformations. Let us stress that MONETA 2.0 [19] also provides a graph representing the protein, but it uses communication pathways and covalent bonds to construct it and the criteria employed are markedly different from those employed by COMMA to construct the PCN.

### Steps d and e. Extraction of communication blocks and communicating segment pairs

COMMA final outputs consist in dynamics-based decompositions of the query protein 3D structure. Two types of decompositions are produced. The protein is divided into: (*i*) communication blocks defined from the PCN, (*ii*) communicating segment pairs defined from secondary structure elements and communication pathways. These two notions are completely new compared to MONETA.

#### Communication blocks

Connected components in an undirected graph are isolated subgraphs. COMMA extracts connected components from the constructed PCN by using depth-first search (DFS) and defines protein communication blocks. Different types of communication blocks are defined, namely clique-based blocks and pathway-based blocks. Clique-based blocks are directly extracted by considering all clique-based edges. Different kinds of pathway-based blocks are defined, either by considering all but very short (≤3 residues) pathways, or by considering pathways longer than a fixed number of residues. An interesting threshold is given by *MPL*_*cut*_ as defined below (see Parameters).

#### Communicating segment pairs

COMMA detects pairs of protein segments that are part of secondary structure elements (SSEs) and that are linked by communication pathways. A SSE is constituted by residues (at least three) that adopt the same secondary structure type. First the algorithm identifies all SSEs contained in the protein structure. Then, it computes, for each pair (*A,B*) of SSEs: (*i*) the proportion *PR*_*AB*_ (resp. *PR*_*BA*_) of residues from *A* (resp. *B*) that are linked by at least a communication path to some residue from *B* (resp. *A*), (*ii*) the number of pairs of residues (*i*^*A*^,*j*^*B*^) of *A* and *B* that are consecutive in a communication path, *Cont*_*AB*_. The residues of *A* and *B* that are linked by at least a communication path constitute a communicating segment pair. The communication strength between the two segments defined from *A* and *B* is calculated as:

(4) $$ S_{AB}=PR_{AB}*PR_{BA}*Cont_{AB}  $$

### Visualisation

COMMA is interfaced with PyMoL [30] to permit the visualisation of the communication blocks and the communicating segment pairs by mapping them on the protein average conformation. COMMA produces PyMoL files (.pml extension) that enable the following representations:

- **Communication blocks:** the residues involved in communication blocks are coloured accordingly. Residues that are not detected in a communication block are coloured in white. Non-covalent interactions between blocks are shown as thick black lines.
- **Communicating segment pairs:** given a pair of SSEs, the residues involved in the communicating segments in these SSEs are highlighted in colours. Pathways-based edges linking residues in the two segments are shown as thick black lines.

### Parameters

COMMA uses several parameters and allows the user to tune them depending on the question asked and on the system studied. However, to allow for a large-scale application of the method, we have implemented automated procedures to set up default values for all parameters.

**Fig. 2:**
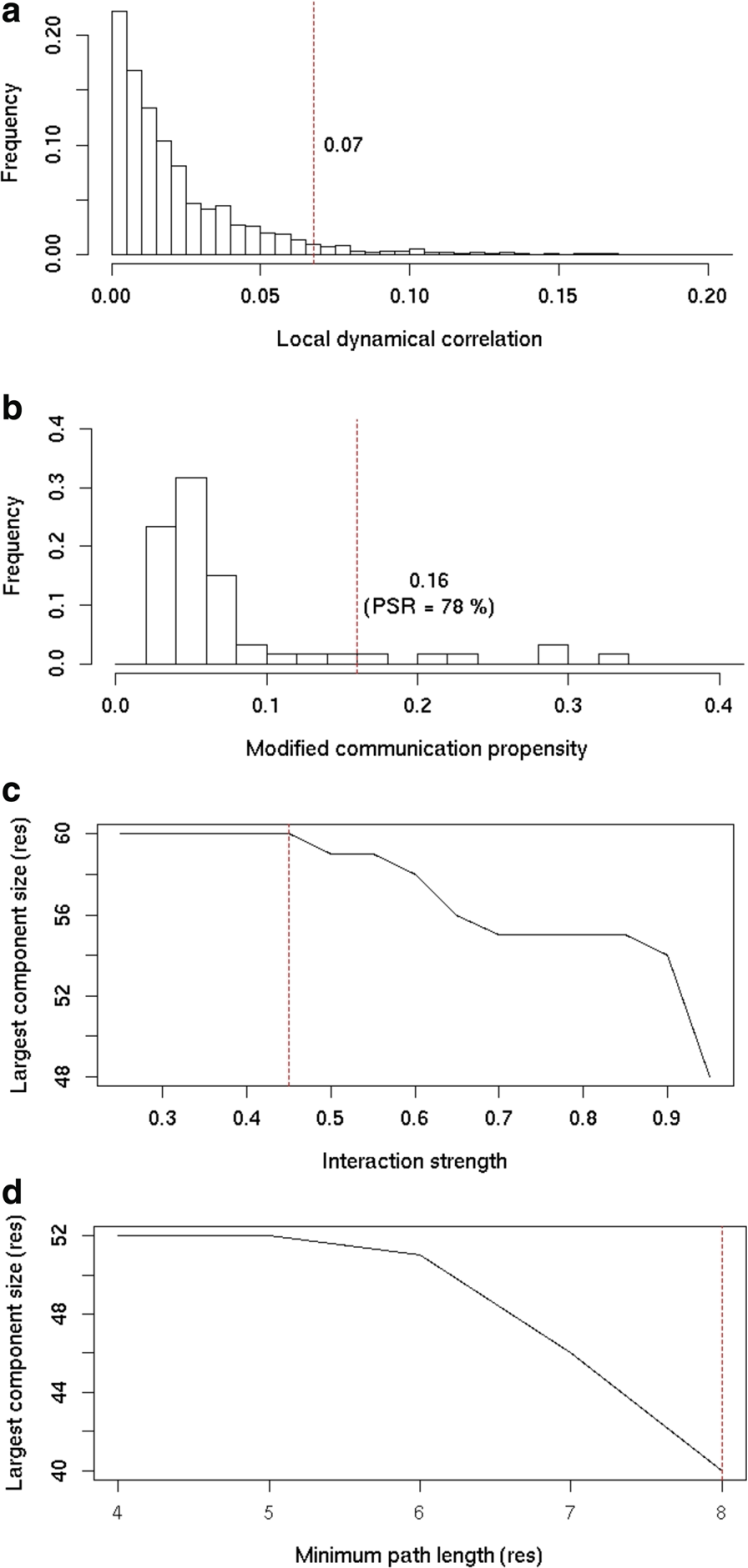
Parameters for protein A. **a** Distribution of the local dynamical correlation (*C* *o* *r* *r*_*LFA*_) values. **b** Distribution of the communication propensity (*CP*) values. **c** Size of the largest connected component (in residues) extracted from the adjacency graph constructed based on non-covalent interaction strengths. **d** Size of the largest connected component (in residues) extracted from the PCN by considering communication pathways with different minimum lengths

$Corr^{LFA}_{\textit {cut}}$ We define the LFA correlation threshold $Corr^{LFA}_{\textit {cut}}$ to delimit protein regions of concerted atomic fluctuations. $Corr^{LFA}_{\textit {cut}}$ is chosen such that 5 % of the values in the *Corr*^*LFA*^ matrix are higher than $Corr^{LFA}_{\textit {cut}}$ (Fig. 2a).

***CP***_***cut***_ We define a cutoff *CP*_*cut*_ to determine whether the communication between two residues is efficient. The strategy employed to set the value of *CP*_*cut*_ is inspired from [31]. Intuitively, neighbouring residues in the sequence forming well-defined secondary structures are expected to communicate efficiently with each other. First, we evaluate the proportion *p*_*ss*_ of residues that are in an *α*-helix, a *β*-sheet or a turn in more than half of the conformations. Then for every residue *i*, we compute a modified communication propensity *MCP*(*i*) as:

(5) $$ MCP(i) = \frac{1}{8} \sum\limits_{\substack{j=i-4\\j \neq i;1 \leq j \leq N}}^{i+4} CP(i,j)  $$

where *N* is the total number of residues. *CP*_*cut*_ is chosen such that the proportion *p*_*ss*_ of *MCP* values are lower than *CP*_*cut*_ (Fig. 2b). Any two residues *i* and *j* for which *CP*(*i,j*)<*CP*_*cut*_ are considered to communicate efficiently.

***INT***_***cut***_ We define a threshold value *INT*_*cut*_ to filter out non-covalent interactions that are not relevant. For this, an adjacency graph is constructed from the *INT* matrix by considering different cutoff values, ranging from 0.25 to 1, by increments of 0.05, and the size of the largest connected component is computed (Fig. 2a). *INT*_*cut*_ is the largest interaction strength for which the size of the largest component is maximal [32] (Fig. 2c).

***MPL***_***cut***_ We define a threshold *MPL*_*cut*_ to discriminate between short and long paths. For this, connected components are extracted from subgraphs of the PCN. The subgraphs are defined by considering pathway-based edges that are derived from communication pathways comprising at least *n* residues, *n* ranging from 4 to 8. *MPL*_*cut*_ is chosen as the minimum path length for which we observe the largest reduction of the size of the largest connected component (Fig. 2d).

### Proteins studied

We applied the COMMA method to three archetypal proteins: (*i*) the B domain of staphylococcal protein A (PDB id: 1BDD, residues 1-60, NMR), a highly stable protein, (*ii*) the DNA-binding domain of the human tumour suppressor protein p53 (PDB id: 2XWR, chain A, residues 89-293, 1.68Å resolution), a highly flexible protein, (*iii*) the cytoplasmic region of the receptor tyrosine kinase KIT (PDB id: 1T45, residues 547-935, 1.90Å resolution), an allosterically regulated protein.

### Molecular dynamics simulations

The same molecular dynamics protocol was applied to all studied systems. More details on the MD trajectories of the wild-type KIT and its oncogenic mutant D816V can be found in [33].

#### Set up of the systems

The 3D coordinates for the studied proteins were retrieved from the Protein Data Bank (PDB) [34]. All crystallographic water molecules and other non-protein molecules were removed. The structure of the DNA-binding domain of P53 contains a bound zinc ion. At physiological temperature, Zn ^2+^ rapidly dissociates from the protein and the resulting Zn ^2+^-free P53 is folded and stable [35, 36]. Consequently, we removed the zinc ion from the initial PDB structure and simulated P53 in the apo form. The mutated form of KIT was generated by *in silico* substitution of the aspartate (D) in position 816 into a valine (V) using MODELLER 9v7 [37]. All models were prepared using the LEAP module of AMBER 12 [38], with the ff12SB forcefield parameter set: (*i*) hydrogen atoms were added, (*ii*) Na ^+^ or Cl ^−^ counter-ions were added to neutralise the systems charge, (*iii*) the solute was hydrated with a cuboid box of explicit TIP3P water molecules with a buffering distance up to 10Å. The environment of the histidines was manually checked and they were consequently protonated with a hydrogen at the *ε* nitrogen. The details of structure preparation and solvent models are given in Additional file 1: Table S1.

#### Minimisation, heating and equilibration

The systems were minimised, thermalised and equilibrated using the SANDER module of AMBER 12. The following minimisation procedure was applied: (*i*) 10,000 steps of minimisation of the water molecules keeping protein atoms fixed, (*ii*) 10,000 steps of minimisation keeping only protein backbone fixed to allow protein side chains to relax, (*iii*) 10,000 steps of minimisation without any constraint on the system. Heating of the system to the target temperature of 310 K was performed at constant volume using the Berendsen thermostat [39] and while restraining the solute *C*_*α*_ atoms with a force constant of 10 kcal/mol/Å^2^. Thereafter, the system was equilibrated for 100 ps at constant volume (NVT) and for further 100 ps using a Langevin piston (NPT) [40] to maintain the pressure. Finally the restraints were removed and the system was equilibrated for a final 100-ps run. Backbone deviations obtained after equilibration are smaller than 1.3 Å (Additional file 1: Table S1).

#### Production of the trajectories

For every protein, 2 replicates of 50 ns, with different initial velocities, were performed in the NPT ensemble using the PMEMD module of AMBER 12. The temperature was kept at 310 K and pressure at 1 bar using the Langevin piston coupling algorithm. The SHAKE algorithm was used to freeze bonds involving hydrogen atoms, allowing for an integration time step of 2.0 fs. The Particle Mesh Ewald method (PME) [41] was employed to treat long-range electrostatics. The coordinates of the system were written every ps. Standard analyses of the MD trajectories were performed with the *ptraj* module of AMBER 12.

#### Stability of the trajectories

The simulations of wild-type and mutated KIT were previously shown to have good stability [33]. To assess the stability of the B domain of protein A and of the DNA-binding domain of p53, the C *α* atoms root mean square deviation (RMSD) from the equilibrated structure, the stability of secondary structures and the radius of gyration were recorded along each 50-ns MD simulation replicate (Additional file 1: Figure S1 and Figure S2). The B domain of protein A deviates by no more than 2.2Å (Additional file 1: Figure S1A) from the equilibrated structure and has an average radius of gyration of 10.5 ± 0.1Å (Additional file 1: Figure S1D). p53 DNA-binding domain displays RMSD values in the range 1.5–3.0Å (Additional file 1: Figure S2A) and its radius of gyration values 16.6 ± 0.1Å (Additional file 1: Figure S2D). Secondary structure profiles are highly stable for both replicates of both proteins (Additional file 1: Figure S1B-C and Figure S2B–C). Overall, the evolution of RMSD, secondary structure and radius of gyration shows that protein A and p53 are stable over the 50-ns runs. The systems are fully relaxed after 20 ns (Additional file 1: Figure S1A and Figure S2A). Consequently, COMMA was applied on the last 30 ns of every replicate. COMMA input sets for the three study cases are made of 30,000 conformations.

#### Convergence of the trajectories

To evaluate the convergence of the dynamic properties extracted by COMMA, a convergence analysis [42] was applied to the MD trajectories of the studied systems. The analysis comprises two steps: (*i*) a set of reference conformations are identified, (*ii*) all MD conformations from the trajectory are clustered into corresponding reference groups. Each reference conformation is first picked up randomly and the conformations distant by less than an arbitrary cutoff *r* are binned with it. Then the trajectory is split in two halves and conformations from each half are grouped based on their RMSD from each reference conformation. If the simulation has converged, then each reference cluster should be populated equally from both halves of the trajectory.

The RMSD was computed on the C *α* atoms and the cutoff *r* was empirically chosen so as to get a reasonable number of representative MD conformations, typically between 2 and 7. To reduce the bias resulting from the random choices of the references, the process was repeated 5 times for each analyzed trajectory. The convergence quality of each simulation was measured using a convergence criterion *c* defined as [43]:

(6) $$ c = 1 - \left(\frac{1}{5} \sum\limits_{k=1}^{5} \frac{\#(\text{lone reference conformations})}{\#(\text{reference conformations})}\right)  $$

A lone reference conformation is a reference conformation that is not visited in one half of the trajectory (less that 1 % of the frames in the corresponding reference group). The convergence criterion *c* is comprised between 0 and 1; a value of 1 corresponds to an optimal convergence. All trajectories show good to very good convergence, with values of *c* ranging between 0.6 and 0.9 (Additional file 1: Table S2). This indicates that the conformational sampling furnished by the last 30 ns of each productive MD run is sufficient to apply COMMA.

## Results and discussion

### Communication blocks in KIT protein and its oncogenic mutant

**Fig. 3:**
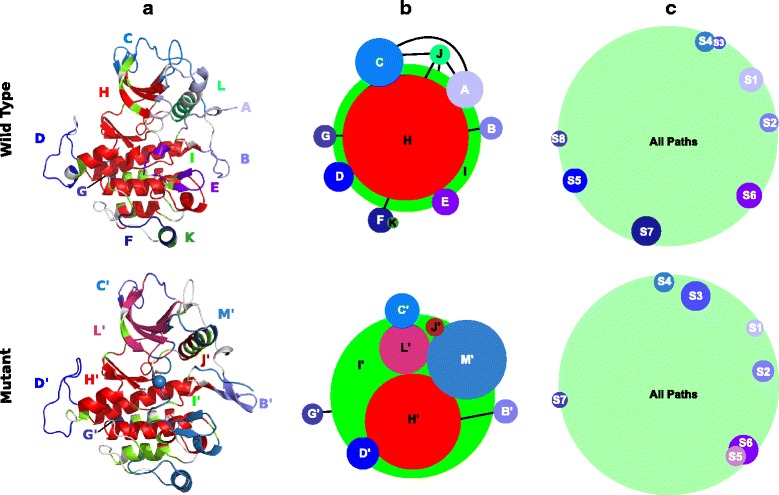
Dynamical architecture of wild-type KIT and the D816V mutant. *On top*. Wild-type protein. *At the bottom*. Mutant protein. *On the left*. The communication blocks identified by COMMA are mapped onto the average conformation represented as a cartoon. The mutation site is represented by a sphere (at the bottom). The protein residues are coloured according to the block they belong to and the different blocks are labelled. See Table 1 for details on the mapping between the two proteins. *In the middle*. Schematic representations of the proteins depicting the communication blocks identified by COMMA and the connections between them. Each block is represented by a round and is labelled. The larger the number of residues in the block, the larger the size of the round. Overlapping blocks share some residues in common. Contacting blocks are connected by covalent bonds. The black links indicate the presence of stable non-covalent interactions between blocks. Notice that non-covalent interactions are formed between blocks that share some residues in common or contact each other, but they are not displayed for a sake of clarity. *On the right*. Schematic representations of the proteins depicting the results obtained from MONETA. The large round in green include all residues involved in some communication pathway. The smaller blocks in blue tones represent independent dynamic segments. The size of the round depends on the number of residues involved (same scaling as for COMMA results)

KIT is a receptor tyrosine kinase of type III implicated in signalling pathways crucial for cell growth, differentiation and survival [44–46]. The mutation of the aspartate located in position 816 to a valine leads to the constitutive activation of the receptor and is associated to mastocytoses and gastrointestinal stromal tumours [47, 48]. It was shown experimentally that the mutation induces long-range effects that lead to a shift in the conformational equilibrium of the kinase away from the auto-inhibited state, resulting in a 536-fold increased activation rate [49]. COMMA was applied to the cytoplasmic region of KIT (331 residues), starting from 2 replicates of 50-ns MD simulations of the wild-type and D816V-mutated proteins [33] (see Methods). The method identified 11 (resp. 9) communication blocks in the wild type (resp. mutant) (Table 1). These blocks reflect the way information is transmitted across the protein structure (see Methods). They were mapped onto the average MD conformations of the wild-type and mutated proteins for visualisation (Fig. 3a). They were also used to derive schematic representations of the two proteins (Fig. 3b).

**Table 1:** Mapping of communication blocks between wild-type KIT and the D816V mutant

Wild type													
Name	A	B	C	D	E	F	G	H	I	J	K	-	-
Size (res.)	22	11	32	16	14	13	11	127	160	9	4	-	-
Mutant													
Name	-	B’	C’	D’	-	-	G’	H’	I’	J’	-	L’	M’
Size (res.)	-	12	20	18	-	-	10	86	186	8	-	35	66
Overlap (%)	-	96	65	76	-	-	95	80	87	71	-	-	-

The overlap *o*_*ij*_ between two blocks *B*_*i*_ and *B*_*j*_, identified in the wild type and in the mutant, is evaluated as: *o*_*ij*_=2∗*#*(*B*_*i*_∩*B*_*j*_)/(*#*(*B*_*i*_)+*#*(*B*_*j*_)). Two blocks are defined as counterparts, namely X and X’ if: (*i*) X’ (resp. X) yields the maximum overlap with X (rest. X’) over all blocks in the mutant (resp. wild-type) protein; (*ii*) the overlap is greater than 60%

#### Decomposition of KIT dynamical architecture

KIT communication blocks can be classified according to the structural and dynamical information used to identify them. In the wild type (Fig. 3a–b, on top), blocks A to G (in blue tones) were obtained from independent cliques (see Methods). These blocks represent protein regions whose internal dynamics are independent from each other and from the rest of the protein. Blocks H (in red), I (in green), J (in lime green) and K (in dark green) were obtained from communication pathways, i.e. chains of dynamically correlated residues stabilised by non-covalent interactions (see Methods). Blocks I, J and K were identified by considering all but very short paths while block H comprises only long paths (≥6 residues).

Different types of connections are established between blocks (Fig. 3a–b), namely, from the strongest to the weakest: (*a*) inclusion, e.g. block H is included in block I, (*b*) overlap, e.g. blocks D and I share some residues in common, (*c*) contact, e.g. some residues from blocks B and I are adjacent in the sequence, (*d*) interaction, e.g. some residues in blocks A and C form a stable H-bond or hydrophobic contact. We observed that two blocks that share residues or contact each other (types *a, b, c*) are also connected by non-covalent interactions (type *d*).

The architecture of KIT is composed of a core of long-range communicating residues forming block H, that represents more than one third of the protein (Table 1). This core spans the two lobes of the protein and covers most of the enzymatic site (Fig. 3a–b, on top). It is extended by a layer of short-range communicating residues contained in block K and is connected to several much smaller blocks. These small blocks establish few connections between them. However an interconnected set of small blocks (A, C, and J) can be detected, that is constituted by residues from the N-terminal lobe and represents about 20 % of the protein.

#### Comparison of wild-type and mutated KIT

The communication blocks identified by COMMA in wild-type and mutated KIT were compared. The pairs of blocks from the two proteins that are constituted in large part by the same residues were identified (Table 1). Overall, the composition of the blocks and their connections can vary substantially upon mutation (Fig. 3b). Specifically, block M’ (in sky blue) of the mutant comprises most of the residues constituting blocks A, E and F in the wild type. Let us stress that the mutational position 816 is located in block E of the wild type protein and in block M’ of the mutant (indicated as a sphere on Fig. 3a, at the bottom). Interestingly, the protein regions comprised in block M’ were recently highlighted as forming an allosteric network in Src kinase [50]. In addition to these changes, COMMA detected three long-range communication blocks in the mutant (in red tones) instead of one in the wild type. Block H’ (in red) is 1.5 times smaller than block H. Some residues from the N-lobe that were included in block H now form the disjoint block L’ (in raspberry). The residues forming block J’ (in firebrick) communicate at longer range than the residues forming block J in the wild type. These three blocks H’, J’ and L’ are included in block I’, which is slightly bigger than I. Consequently, the mutation induces a complete reshaping of communication blocks in KIT, characterised by a reorganisation of the hierarchy between long-range and short-range communicating residues and the merge of three clique-based blocks.

#### Comparison with other classifications

The definition of KIT communication blocks provided by COMMA can be compared with the definition of KIT regulatory regions reported in the literature [51–54]. Blocks B, C, D, E, F and L partially match the JM-Switch (JMS), the JM-Zipper (JMZ), the kinase insert domain (KID), the A(ctivation)-loop, the substrate-binding platform (helix G) and the C-helix respectively (Additional file 1: Figure S3A). Block A contains the JM-Proximal (JMP) and the glycine-rich loop (P-loop). The blocks can also be evaluated based on the flexibility profile of the residues they contain. Pathway-based blocks tend to contain rather rigid residues while clique-based blocks are highly flexible (Additional file 1: Figure S3B). From a secondary structure perspective, residues in pathway-based blocks tend to form stable secondary structures whereas residues in clique-based blocks are in solvent-exposed loops (Additional file 1: Figure S3C). We observed that these trends are general among the proteins we studied. These observations show that the identification of communication blocks by COMMA correlates positively with protein residue classifications based on the literature, on rigidity/flexibility or on secondary structures. Furthermore, COMMA enables to go beyond such classifications by providing a more precise dissection of the protein’s dynamical architecture.

#### Comparison with MONETA

COMMA results were compared to those obtained with MONETA 2.0 (Fig. 3c). MONETA identifies independent dynamic segments and communication pathways from all-atom MD simulations [19], which are similar to the independent cliques and communication pathways identified by COMMA (Fig. 1, boxes 2 and 3). However, COMMA exploits these components for further analysis (Fig. 1, boxes 4, 5 and 6) in a way that is completely different from MONETA [19]. Figure 3c depicts schematic representations of the dynamic segments and communication pathways detected by MONETA in KIT. The green round corresponds to the ensemble of residues involved in some path (representing 90 % of the protein). The rounds in blue tones represent dynamic segments. These components are substantially different from the communication blocks identified by COMMA (Fig. 3b) and MONETA does not characterise the connections between them. From this comparison, it is clear that COMMA brings additional information on the definition and arrangement of the protein’s dynamical architecture building blocks, compared to MONETA.

MONETA previously permitted to put in evidence a crucial communication pathway in wild-type KIT that links the A-loop and the JMS through residue D792 from the catalytic loop [25]. The path was disrupted upon D816V mutation. In COMMA representation of wild-type KIT (Fig. 3, on top), all residues participating in this path are contained in the long-pathway based block H (in red), from D792 in the catalytic loop to V559 in the JMS. By contrast, in the mutant (Fig. 3, at the bottom), D792 is contained in the pathway-based block I’ (in green) but not in block H’ (in red), indicating that this residue is involved in shorter communication pathways compared to the wild type, and that no pathway goes from D792 to the JMS. COMMA results are thus in agreement with those obtained by using MONETA. Moreover, by identifying communication blocks, COMMA enables to pinpoint other long pathways that are interrupted in the mutant. Specifically, the fact that the long-pathway-based block H in the wild type is divided in H’ and L’ in the mutant is associated to a disruption of the communication between residue N655 and residues I653, H651 and K807. Interestingly, these residues were shown to form a network of interactions (called ‘molecular brake’) crucial for the stability of the inactive conformation of tyrosine kinases [55]. Consequently, COMMA analysis permits to put in evidence a deleterious effect of the activating D816V mutation on this ‘molecular brake’ which was not previously detected.

This analysis illustrates how COMMA can help dissect a protein 3D structure from a dynamical perspective and characterise the effect of a deleterious mutation on the structural dynamics of a protein. The information provided by COMMA was found in agreement with the previous findings on KIT allosteric communication. It further allows a more systematic assessment of the differences between two proteins or two states of the same protein and permits to pinpoint with high precision regions or residues instrumental in the establishment or alteration of the protein communication.

### Communicating segment pairs in protein A

The B domain of protein A (BdpA) from *Staphylococcus aureus* is a small *α*-helical protein. It comprises 60 residues arranged in three helices, namely H1 (residues 10-19), H2 (residues 25-37) and H3 (residues 42-56), linked by two turns, namely T1 (residues 20-24) and T2 (residues 38-41). The fast-folding kinetics of protein A have been extensively characterised through experiments and computer simulations [56–60], enabling to establish the following statements: (*i*) the isolated H3 has a higher stability and helical content compared to the two other helices, (*ii*) H2 and H3 form a stable or marginally stable intermediate, (*iii*) H1 is docked in the rate limiting step.

COMMA was used to identify communicating segment pairs in BdpA (60 residues). For this, we performed 2 replicates of 50-ns MD simulations, starting from an average nuclear magnetic resonance (NMR) structure (see Methods). By analysing the MD trajectories, COMMA detected five stable secondary structure elements (SSEs) in the protein: three *α*-helices formed by residues 5-18, 25-37 and 39-55 and two turns formed by residues 2-4 and 56-59. We focus here on the three *α*-helices, which match well the experimentally-defined helices H1, H2 and H3. Three pairs of communicating segments were identified between H1/H2, H1/H3 and H2/H3 (Fig. 1, box 6). The communication strengths (computed as the product of the proportions of residues involved in communication pathways linking the two segments multiplied by the number of pairs of residues directly linked by a pathway, see Methods) for these pairs are 0.5, 1.1 and 4.1 respectively. The significantly higher strength of the segment pair corresponding to H2/H3 is the result of a larger number of residues involved in the communication and a larger number of direct links (5 versus 2 and 3, shown as black lines on Fig. 1, box 6). Let us remind that a direct link is a pair of residues from the two communicating segments that are consecutive in a communication path (see Methods). Moreover, one can observe that the communicating segments of H1 cover a significantly smaller portion of the helix compared to the segments of H2 and H3. The communication blocks identified in protein A also show that the residues of H1 are involved in shorter paths compared to H2 and H3 (Fig. 1, box 5). These observations are in agreement with the experimental evidence that H1 docks to a stable assembly of H2 and H3 during the folding process. Let us stress that this result could not be obtained by simply analysing non-covalent interactions along the MD trajectories: there are 8, 4 and 8 interactions for the H1/H2, H1/H3 and H2/H3 pairs. This emphasises the importance of the notions of communication propensity and communication pathways in our analysis.

### The role of pathway length and interaction type in p53 communication

**Fig. 4:**
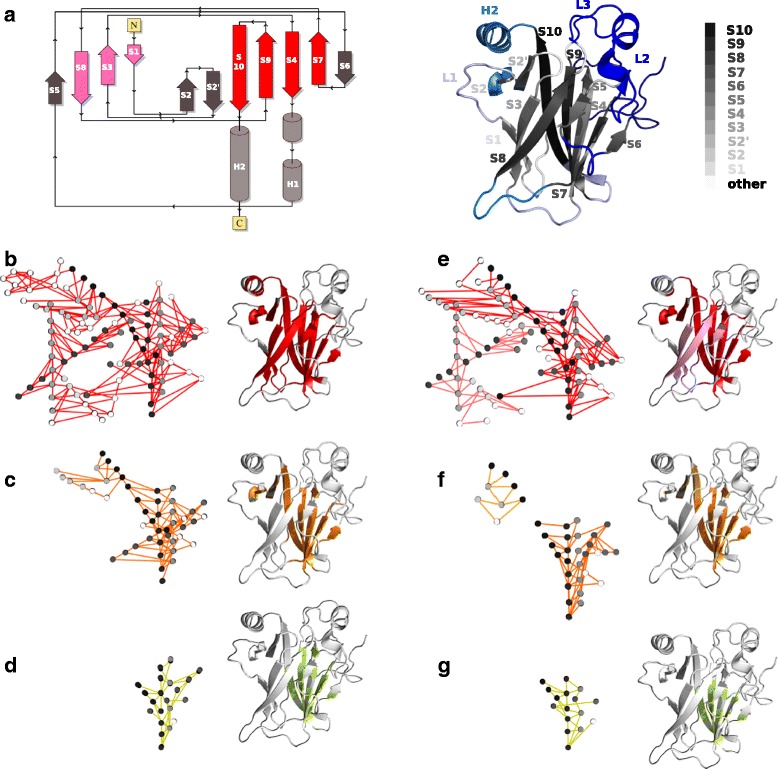
Influence of pathway length and interaction type on P53 DBD communication. **a** 2D topology diagram (*on the left*) and 3D structure (*on the right*) of p53 DBD. The diagram was taken from PDBsum [71] and the colours were modified to put in evidence the S-type immunoglobulin-like fold of p53 DBD: the first *β*-sheet is in pink, the second *β*-sheet is in red. The 3D structure (average MD conformation) is represented as a cartoon, where the 11 *β*-strands of the protein are coloured in grey tones and labelled. The clique-based communication blocks identified by COMMA are colored in blue tones. **b**–**g** Pathway-based communication blocks identified by COMMA by using information from all non-covalent interactions (**b**–**d**) or only interactions involving side chains (**e**–**g**), and by considering only pathways longer than 3 (**b, e**), 5 (**c, f**) or 7 (**d, g**) residues. The communication blocks are represented as subgraphs in the PCN (*on the left*) and are mapped on the average MD conformation (*on the right*). The edges on the subgraphs and the residues on the 3D structure are coloured according to the communication blocks they belong to. The nodes in the subgraphs are coloured in grey tones, indicating the *β*-strand they belong to

The tumour suppressor p53 is a transcription factor regulating a wide range of genes involved in DNA repair, apoptosis, senescence and metabolism [61–63]. The p53 protein plays a crucial role in conserving the stability of the genome and preventing genomic mutation [64]. The loss of p53 tumour suppressor function is associated with cancer [65]. The sequence of p53 can be divided into an N-terminal transactivation domain, a DNA-binding core domain (DBD), a tetramerisation domain and a C-terminal regulatory domain [66]. The DBD is intrinsically unstable and thus highly susceptible to oncogenic mutations [67]. The three-dimensional structure of the DBD comprises two antiparallel *β*-sheets, characteristic of the immunoglobulin-like *β*-sandwich fold (Fig. 4a, topology diagram on the left). In total, it contains 11 *β*-strands and 2 *α*-helices linked by flexible loops (Fig. 4a, see labels on the right). The dynamical architecture of p53 DBD (199 residues) was characterised by COMMA, starting from 2 replicates of 50-ns MD simulations (see Methods). We investigated the evolution of the pathway-based communication blocks identified by COMMA when varying the minimum length of the pathways considered and the type of non-covalent interactions used to construct them (Fig. 4).

#### Hierarchical description of p53 communication

The ensemble of all but very short (≤ 3 residues) communication pathways identified in p53 yielded one communication block (Fig. 4b, in red), representing about 50 % of the protein residues. This block comprises the 11 *β*-strands of the protein, some residues from the loops that frame them and a portion of the helix H2. The edges of the corresponding subgraph show that communication pathways go along individual *β*-strands (the nodes coloured in the same grey tone belong to the same *β*-strand) and also cross them. The edges linking different *β*-strands reflect well the interactions that stabilise the two *β*-sheets of the protein. Filtering out pathways smaller than 6 residues yields a communication block twice as small (Fig. 4c, in orange). The *β*-strands S1, S3 and S8 that form the first *β*-sheet (Fig. 4a, in pink) are completely absent from the block, as well as helix H2. The block is further reduced by two times when keeping only very long (≥ 8 residues) pathways (Fig. 4d, in lime green). Only a portion of the second *β*-sheet, composed of S4, S7, S9 and S10 (Fig. 4a, in red), remain in the block. This region can be viewed as the communication core of the protein.

#### Influence of non-covalent interaction type

Secondary structure units (e.g. *β*-sheets) are stabilised by H-bonds formed between backbone atoms (e.g. from parallel or anti-parallel *β*-strands). We analysed the impact of disregarding information from these interactions on p53 DBD communication. Only interactions involving side chain atoms were retained to construct communication pathways and the corresponding communication blocks were extracted (Fig. 4e-g). The obtained subgraphs show a significantly reduced number of edges linking different *β*-strands. This result is expected owing to the nature of *β*-sheets. More surprisingly, however, the smaller number of edges minimally impacts the communication within each *β*-sheet. This indicates that numerous interactions are established within the *β*-sheets, other than backbone-backbone H-bonds. By contrast, the loss of these interactions is determinant for the communication between the two *β*-sheets and results in each of them being detected as an isolated communication block (Fig. 4e, in red and pink). Two communication blocks are also detected when pathways smaller than 6 residues are filtered out (Fig. 4f, in orange and yellow-orange), instead of one with all interactions (Fig. 4c). This is due to backbone-backbone interactions being lost within S10 and between S10 and S9. The communication core of the protein, obtained from very long pathways (Fig. 4g), is slightly smaller than when considering all interactions (Fig. 4d), due to missing interactions involving S7.

This analysis unveiled the hierarchical roles played by the different structural units (i.e. *β*-sheets) of the p53 DBD in the protein’s dynamical architecture. Specifically, the residues constituting the first *β*-sheet communicate at shorter range than those constituting the second *β*-sheet. Furthermore, it showed the preponderant role of backbone-backbone interactions in establishing communication between the two *β*-sheets. These results illustrate how COMMA can be employed to contrast different protein regions from a dynamical point of view and to investigate the molecular determinants of protein communication at a precise level.

### Comparison of protein A and p53

The B domain of protein A and p53 DBD represent two archetypal proteins in terms of thermodynamic and kinetic stability. While the latter unfolds at just above physiological temperature [68], the former presents fast and stable folding [56]. Moreover, BdpA is composed of three helices while p53 DBD mainly contains *β*-sheets. Consistently, our analyses of the two proteins show very different results. COMMA identified 2 very small clique-based communication blocks in BdpA, corresponding to the two extremities and representing 13 % of the protein residues. By contrast, the clique-based communication blocks identified in p53 DBD represent almost 60 % of the protein (Fig. 4a, on the right and in blue tones). They encompass all residues involved in the interaction with DNA, namely the loops L1, L2 and L3 and the helix H2, which adopt variable conformations in the available experimental structures of p53 DBD [69]. COMMA also enabled to characterise the evolution of pathway-based communication blocks when varying the minimum communication pathway length. The communication core of BdpA, defined based on very long (≥ 8 residues) pathways, comprises full-length helix H3 and some residues from H1 and H2 (Fig. 1, box 5, in yellow). This is consistent with experimental evidence showing that H3 is the most stable helix among the three [60]. p53 DBD presents a strikingly different dynamical behaviour, with a communication core composed of residues from different *β*-strands that form the first *β*-sheet (Fig. 4d). Progressively filtering out communication pathways with increasing length results in residues, first from the loops that frame the *β*-strands, then from the extremities of the *β*-strands, to be excluded from the communication block (Fig. 4b–d). Notice that the length of the pathways does not depend on the length of the *β*-strands, i.e. longer *β*-strands do not exhibit longer paths. These observations on BdpA and p53 DBD support the utility of COMMA to compare proteins of very different natures in a straightforward way.

### The importance of the conformational sampling

The results obtained from COMMA directly depend on the extent and quality of sampling in the input conformational ensemble. In the case of MD trajectories, the user must carefully check that they have converged before proceeding through COMMA analysis. In the present work, we have performed COMMA analysis on the conformational ensemble generated during the last 30 ns of two 50-ns MD replicates for each studied system. We have assessed the stability of the studied systems in the chosen force field description (Additional file 1: Figure S1A and Figure S2A) and the convergence of the MD trajectories (Additional file 1: Table S2). We have also applied COMMA to the single trajectories and have obtained similar results (Additional file 1: Table S3 and Table S4). This indicates that our results are reproducible and robust to limited variations of the conformational ensemble. Another important aspect is the number of input conformations. In order to get statistically significant results, in particular for the principal component analysis, the number of conformations shall in principle be larger that the number of degrees of freedom of the system studied. In the examples of application reported here, we have characterised the internal dynamics of three proteins on relatively short simulation times (replicates of 50 ns). Consequently, we have illustrated how COMMA can reveal the dynamical dimension of a 3D structure representing a particular macrostate of the protein. Nevertheless, the utility of COMMA is not limited to such type of analysis and the tool can be applied to atomistic simulations sampling large conformational changes.

### Related tools

As noted in the introduction, a number of previously developed methods are dedicated to the analysis of the dynamical behaviour of proteins and their inter-residue communication [10, 11, 16, 17, 20]. These tools however typically consider only dynamical correlations or/and non-covalent interactions, whereas COMMA combines four different dynamical properties in a unified framework (Table 2). Moreover COMMA describes communication at different levels, from individual residues to the whole dynamical architecture of the protein. In particular, the identification of communicating pairs of secondary structure elements is a unique feature of our method (Table 2). Finally, COMMA, which uses MDTraj Python package [70], does not depend on a particular MD package and can handle most popular formats used in the protein structural dynamics community.

**Table 2:** Comparison between different methods to analyse the dynamical behaviour of proteins and their inter-residue communication

	COMMA	Bio3D [11]	GSATools [16]	PyInteraph [10]	PSN-ENM [20]	Taylor et al. [17]
Software availability	✓	✓	✓	✓	-	-
Open source	✓	✓	✓	✓	-	-
Dependencies	MDtraj, Eigen and Numpy python packages	R, Muscle	GNU, Scientific Library, GROMACS	Python, Pymol	-	-
Programming language	C++, Python	R	C	Python	-	-
Input trajectory formats	AMBER, GROMACS, NAMD, CHARMM...	GROMACS (.dcd)	GROMACS	AMBER, GROMACS, NAMD, CHARMM...	-	-
Dynamical properties:						
non-covalent interactions	✓	-	-	✓	✓	✓
inter-residue distances	✓	-	-	-	-	✓
secondary structures	✓	-	-	-	-	-
dynamical correlations	✓(PCA, LFA, CP)	✓(ENM-NMA, PCA)	✓(between frames)	-	✓(ENM-NMA)	✓(MI)
Description levels:						
residue	✓	-	✓	✓	✓	✓
secondary structure	✓	-	-	-	-	-
region/domain	✓	✓	✓	-	-	✓
protein	✓	✓	✓	✓	✓	✓
Outputs:						
protein network	✓	-	✓	✓	✓	✓
communicating regions	✓(pathway- and clique-based blocks)	✓(dynamic domain, correlation network)	✓(functional fragments)	-	-	✓(communities)
communicating segment pairs	✓	-	-	-	-	-
functional domains	-	-	✓	-	-	✓
pathways	✓	-	✓	✓	✓	✓

The technical characteristics and functionalities of COMMA and of five state-of-the-art methods are reported. The PSN-ENM method [20] and the method proposed by Taylor et al. [17] are not implemented as software

## Conclusion

We provide to the community a fully automated tool for analysing conformational ensembles of proteins. The power of the COMMA method resides in the fact that it computes a number of dynamic properties of a protein at the residue level and integrates them in a unified framework to dissect the protein dynamical architecture by identifying its building blocks and the connections between them. COMMA permits to enrich the knowledge of a protein structure by bringing precise, complete and synthetic information on/from its internal dynamics. Moreover, the automatic set up of the parameters implemented in COMMA allows for an adapted modelling of the system under study and to contrast the roles of the different protein regions. COMMA can advantageously complement classical analyses of protein structures and simulations and help look at proteins as dynamical biological objects with a new eye.

## Availability and requirements

**Tool name**: COMMA**Download site**: www.lcqb.upmc.fr/COMMA**Operating system(s)**: Platform independent**Programming language(s)**: C++ and Python 2.7**External tools**: PyMol **Other requirements**: MDTraj, Eigen and Numpy python packages

## Additional file

Additional file 1 **Supplementary Materials.**
