# Supplementary material for: Dissecting protein architecture with communication blocks and communicating segment pairs
Source: BMC Bioinformatics. 2016 Jan 20;17(Suppl 2):13. doi: 10.1186/s12859-015-0855-y (PMC4959365; doi:10.1186/s12859-015-0855-y)
Supplement: Supplementary file 1 — Supplementary Materials. [file 12859_2015_855_MOESM1_ESM.pdf]

Supplementary Materials

	Protein A	P53	KIT WT	KIT MU
Total charge of counter-ions	+2	-3	+1	0
Water box dimensions (Å^3)	75x49x49	76x73x64	77x73x81	77x73x81
Number of water molecules	4 174	8 215	13 195	13 197
Total number of atoms	13 477	27 755	44 870	44 879
Deviation after equilibration (Å)	0.83	0.65	1.29	1.13

Table S1 **MD preparation and equilibration details. The counter-ions employed to neutralize the systems are Na^+ and Cl^-. Root mean square deviations were computed on the backbone atoms of the equilibrated conformations versus the initial template.**

	Protein A		P53		KIT WT		KIT MU	
Replicate	1	2	1	2	1	2	1	2
Cutoff (Å)	1.5	2.2	2.3	2.3	2.5	2.5	2.5	2.5
# reference conformations	2-3	2-6	2-4	2-5	4-7	5-7	2-4	5-6
Convergence criterion c	0.9	0.8	0.9	0.9	0.6	0.4	0.7	0.6

Table S2 **Convergence analysis of the two MD replicates of each studied system. The analysis was applied 5 times on the last 30 ns of every productive run. The convergence criterion c was calculated as described in Methods.**

	pathway-based CB					clique-based CB
	$l \geq 4$	$l \geq 5$	$l \geq 6$	$l \geq 7$	$l \geq 8$	All
**P53**						
all	1	1	1	1	1	5
sim1	3 (55)	3 (61)	1 (84)	1 (75)	1 (88)	8 (78)
sim2	1 (95)	1 (82)	1 (87)	1 (80)	1 (59)	8 (91)
**Protein A**						
all	1	1	1	1	1	2
sim1	1 (100)	1 (100)	1 (98)	1 (100)	1 (98)	2 (100)
sim2	1 (88)	1 (82)	1 (82)	1 (80)	1 (78)	2 (88)
**KIT WT**						
all	3	1	1	1	1	7
sim1	3 (94)	1 (93)	1 (88)	1 (96)	1 (96)	6 (76)
sim2	2 (92)	1 (95)	2 (90)	1 (87)	1 (80)	6 (58)
**KIT MU**						
all	1	2	3	2	2	5
sim1	2 (76)	2 (70)	2 (31)	1 (52)	1 (51)	5 (90)
sim2	1 (96)	2 (94)	3 (90)	2 (98)	2 (100)	6 (68)

Table S3 Reproducibility of communication blocks over the MD replicates of each studied system. The numbers of pathway-based and clique-based communication blocks (CBs) identified by COMMA when applied to the whole conformational ensemble and to the individual MD trajectories are indicated. For pathway-based CBs, different minimum pathway lengths l (in residues) are considered. The overlap (in percentages of residues) between the CBs identified from the first (resp. second) MD replicate and those identified from the whole conformational ensemble are indicated in parentheses.

	all	sim1	sim2
H1-H2	0.5	0.1	0.3
H1-H3	1.1	1	0.3
H2-H3	4.1	4.1	2.6

Table S4 Reproducibility of communication strengths between secondary structure elements (SSEs) in Protein A. The communication strengths (computed as the product of the proportions of residues involved in communication pathways linking the two segments multiplied by the number of pairs of residues directly linked by a pathway, see Methods) between pairs of helices (H1, H2, H3) are reported for the whole conformational ensemble and for each individual replicate. The order of communication strengths is the same in the three analyses.

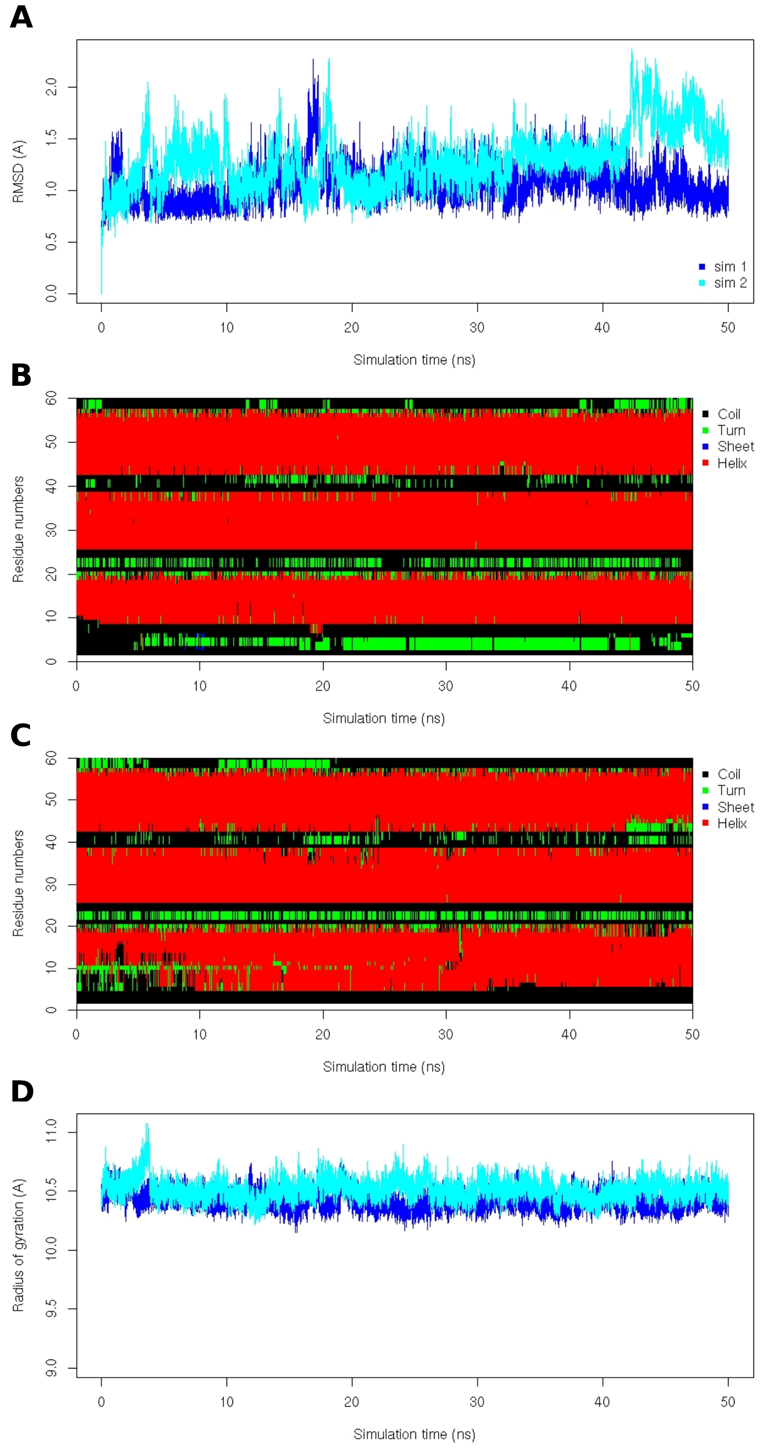

Figure S1 Stability analysis of the B domain of Protein A over 50 ns of MD simulations. A) RMS deviations from the equilibrated structure, computed on the Cα atoms. The first and second MD replicates are in blue and cyan. B,C) Secondary structures recorded over simulation time for the first (B) and second (C) MD replicates. D) Radius of gyration.

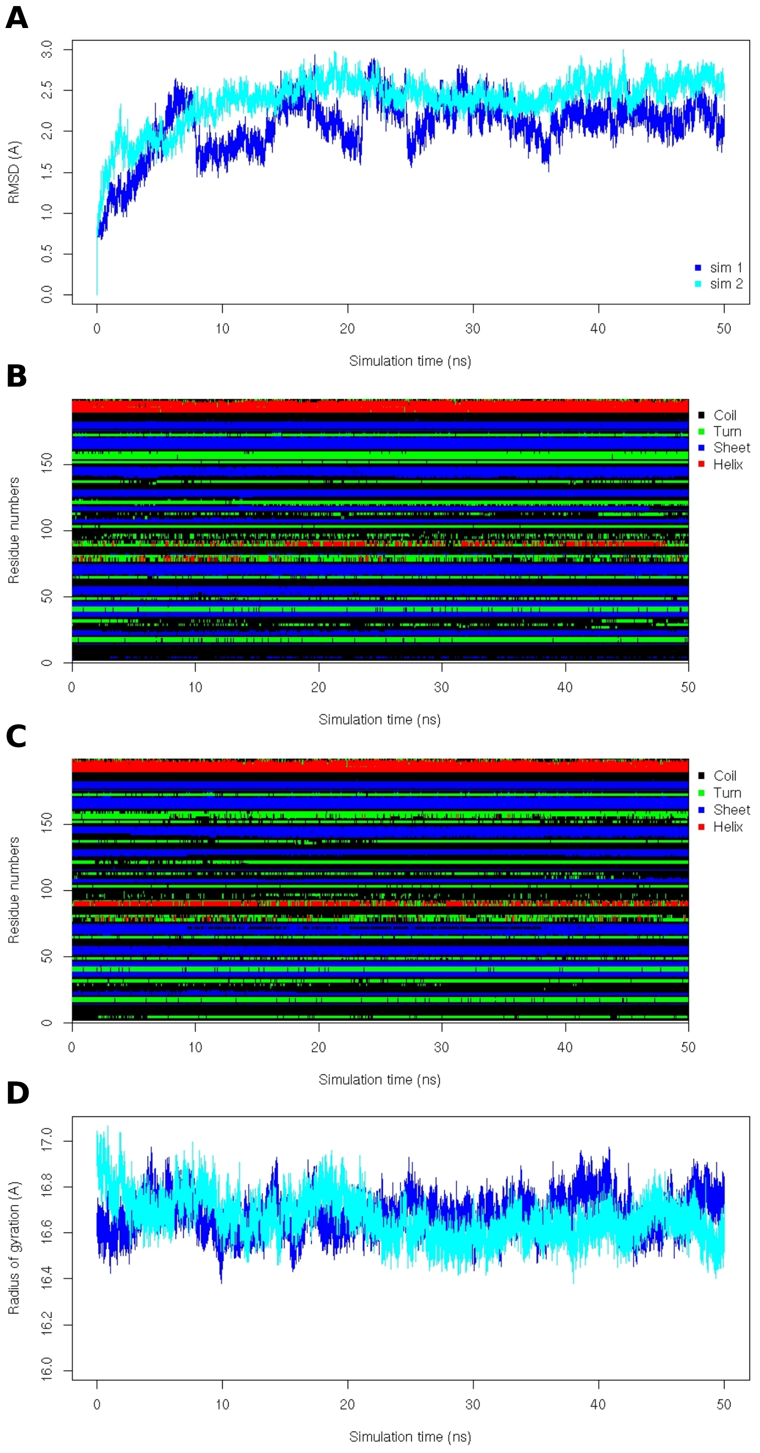

Figure S2 Stability analysis of P53 DNA-binding domain over 50 ns of MD simulations. A) RMS deviations from the equilibrated structure, computed on the Cα atoms. The first and second MD replicates are in blue and cyan. B,C) Secondary structures recorded over simulation time for the first (B) and second (C) MD replicates. D) Radius of gyration.

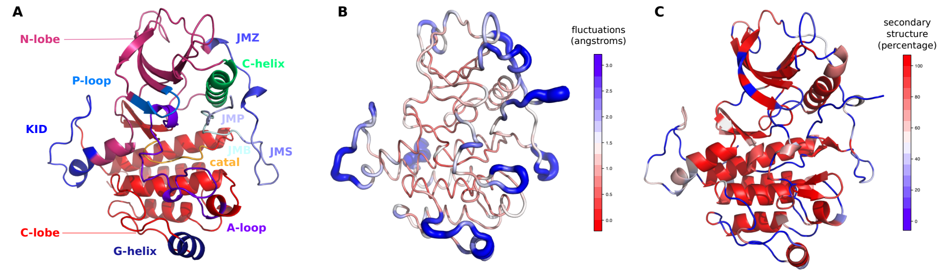

Figure S3 Classifications of KIT residues based on the literature, the rigidity/flexibility and the secondary structures. The crystallographic structure 1T45 (**A**) or the MD average conformation (**B-C**) of KIT cytoplasmic region is represented as a cartoon. (**A**) KIT regulatory regions defined in the literature are highlighted by colours: the JM-Proximal (JMP, residues 547-552) in light blue, the JM-Binder (JMB, residues 553-559) in light cyan, the JM-Switch (JMS, residues 560-570) in slate, the JM-Zipper (JMZ, residues 571-581) in sky blue, the glycine-rich loop (P-loop, residues 596-601) in marine, the C-helix (residues 631-647) in lime green, the catalytic loop (catal, residues 790-797) in orange, the activation loop (A-loop, residues 810-835) in purple and the helix G (residues 877-885) in dark blue. The N-terminal lobe is coloured in raspberry, the kinase insert domain (KID) in blue and the C-terminal lobe in red. (**B**) The per-residue atomic fluctuations (in Å) computed over the MD conformational ensemble are indicated by colours ranging from red (low fluctuations) through white to blue (high fluctuations) and by the size of the tube (the larger the tube the higher the fluctuations). (**C**) The percentages of conformations in which every residue is in a secondary structure element (either α-helix, β-sheet or turn) are indicated by colours ranging from blue (low percentage) through white to blue (high percentage).
